# Familial risk associated with lung cancer as a second primary malignancy in first-degree relatives

**DOI:** 10.1186/s12885-022-10149-7

**Published:** 2022-10-12

**Authors:** Jianguang Ji, Jan Sundquist, Kristina Sundquist, Guoqiao Zheng

**Affiliations:** 1 Center for Primary Health Care Research, Lund University/Region Skåne, Jan Waldenströms gata 35, 205 02 Malmö, Sweden; 2 Department of Family Medicine and Community Health, Department of Population Health Science and Policy, Icahn School of Medicine at Mount Sinai, New York, USA; 3 Center for Community-Based Healthcare Research and Education (CoHRE), Department of Functional Pathology, School of Medicine, Shimane University, Matsue, Japan

**Keywords:** Familial clustering, Multiple primary cancer, Cumulative incidence, Relative risk

## Abstract

**Background:**

Aggregation of lung cancer (LCa) in family members is well-documented. However, little is known on the familial risk of LCa when first-degree relatives (FDRs, parents or siblings) are diagnosed with LCa as a second primary malignancy (LCa-2). We aimed to investigate whether and to what extent a family history of LCa-2 was associated with an increased LCa risk.

**Methods:**

In this Swedish national cohort we identified 127,865 individuals who had one FDR affected by LCa as a first primary cancer (LCa-1) and 15,490 individuals who had one FDR affected by LCa-2, respectively. We then estimated relative risk (RR) of LCa using those without cancer family history as reference.

**Results:**

The number of LCa-2 has been increasing annually and rather similarly in men and women in the last decade. Familial RR of LCa was 1.96 (95%, 1.85–2.07) for LCa-1 family history and 1.89 for LCa-2 (1.62–2.21). Risk was especially high when FDR was diagnosed with early-onset LCa-2 and when siblings were affected by LCa-2. The RR was 1.53 (1.10–2.12) when LCa-2 in FDR was diagnosed within 26 months after first primary cancer, and it increased to 2.16 (1.62–2.90) when LCa-2 was diagnosed between 74 to 154 months. Higher risk was observed for first primary cancer of the ovary (4.45, 1.85–10.7), nervous system (3.49, 1.45–8.38), upper aerodigestive tract (2.83, 1.78–4.49) and cervix (2.55, 1.41–4.61), and for non-Hodgkin lymphoma (3.13, 1.57–6.27).

**Conclusions:**

LCa risk is associated with diagnosis of LCa-2 in FDR to a similar degree as LCa-1 in FDRs.

**Supplementary Information:**

The online version contains supplementary material available at 10.1186/s12885-022-10149-7.

## Background

With the rise in cancer incidence and the continuous improvement in treatment, more and more cancer patients can survive longer time. At the same time, the number of patients having a second primary cancer is increasing. As a common first primary cancer and leading cause of cancer-related death [1], lung cancer (LCa) also accounts for large number of second primary cancer following other cancers [2, 3]. Therefore, more and more people will have family members diagnosed with LCa as a second primary malignancy (LCa-2). A diagnosis of LCa in family member is associated with approximately two-fold increased personal risk [4–7]. However, very little is known on the familial risk of LCa when first-degree relatives (FDRs, parents or siblings) were diagnosed with LCa-2 since most studies refute the order of the primary LCa in relatives. In addition to the inherited genetic predisposition, the familial aggregation of LCa can be due to the shared environmental and lifestyle-related factors such as smoking among family members [5, 8]. Some factors that lead to diagnosis of LCa-2, such as the treatment from previous malignancy [9–11], cannot be shared among family members. Therefore, we expect that the association of LCa risk with family history of LCa-2 will be different from that with family history of LCa-1. Regarding the same matter, we have observed the lower prostate cancer risk in association of prostate and colorectal cancers diagnosed as second primary cancer in FDRs compared to prostate cancer as first primary cancer [12, 13]. While for breast cancer, we did not identify differed breast cancer risk for family history of breast cancer as first and second primary cancer [14].

By incorporating several Swedish national registers, we aimed to estimate the risk of LCa when FDRs were diagnosed with LCa-2 after other cancers, and compare this familial risk to the risk that was related to family history of LCa-1. Our results will provide the first-hand information on LCa risk prediction for people who have FDRs affected by LCa-2.

## Methods

### Data resources

Multiple Swedish national registers were used in this study and the linkage of them was based on the unique personal identification number that has been replaced by a serial number to preserve confidentiality. Family relationships in FDRs were obtained through the Swedish Multi-Generation Registry. It records all the offspring born since 1932 and individuals still alive since 1961 together with their biological parents. The Swedish Cancer Register, with coverage of over 90%, recorded all incident tumors since 1958 [15]. A total of two million cancers were recorded in the latest version. The seventh International Classification of Disease (ICD-7) was used to identify LCa and other cancers. Information on socioeconomic status, place of residence and death notification were obtained with further linkage to Total Population Register and Cause of Death Register. As information on smoking habit, alcohol consumption and body mass index (BMI) were not available, we used the record of hospitalization due to chronic obstructive pulmonary disease (COPD), alcoholism and obesity from the Swedish National Patient Register as the proxy variables, respectively.

### Study population

**Fig. 1:**
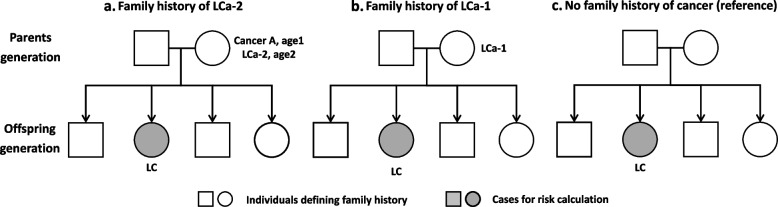
Display of how to analyze familial risk of LCa using a pedigree as an example. Parents and siblings were used to define family history. Cases in the offspring generation were used to estimate risk. In **a**, mother was first diagnosed with cancer A (first primary cancer) at age1 and then LCa-2 at age2. In **b**, mother was diagnosed with LCa-1. In **c**, no first-degree relatives were diagnosed with any cancer. LCa, lung cancer, cancer A, any cancer other than lung cancer, LCa-1, lung cancer as a first primary malignancy, LCa-2, lung cancer as a second primary malignancy

Family history of LCa-2 was defined as LCa diagnosis after other first primary cancer in FDR (parents or siblings). As shown in Fig. 1a, mother was diagnosed with LCa at age2 after cancer A. Family history of LCa-1 was defined as single LCa diagnosis in FDR (Fig. 1b). Individuals without cancer diagnosis in FDR was used as reference group (Fig. 1c).

For the familial risk estimation, we identified individuals who were at risk of first LCa in the offspring generation. We only included those who had a single FDR affected by single LCa-1 or single LCa-2 as the risk groups in order to achieve accurate risk estimation. Therefore, we excluded individuals who had: 1) any FDR affected by multiple primary LCa, 2) any FDR affected by other first primary cancer (except those who further were diagnosed with LCa-2), 3) more than one FDR affected by LCa-1 or LCa-2 and 4) any FDR affected by cancer after LCa-1 or LCa-2. The study population were followed from 1958, birth or immigration, whichever came latest. The follow-up was ended in 2015, first primary LCa diagnosis, death or migration, whichever came earliest.

### Statistical analyses

Annual number of LCa-2 diagnosed from 1958 until 2015 in the cancer registry (both parental and offspring generation) was counted to show the temporal trend of LCa-2 in Sweden.

The relative risk (RR) of LCa in offspring were estimated with Poisson regression adjusting for age (5-year group), gender, periods (5-year group), socioeconomic status (blue-collar worker, white-collar worker, farmer, private business, professional, or other/unspecified) and place of residence (big cities, northern Sweden, southern Sweden and unspecific) as well as hospitalization due to COPD, alcoholism and obesity. We further stratified the risk based on the age at diagnosis of LCa in FDRs (≤ 60 and > 60 years), family relationship (parent–offspring or siblings), the first primary cancer sites in FDRs and time interval (quartiles) between two cancers in FDRs. The cumulative incidence across age and the 95% confidence interval (CI) were calculated, considering death and diagnosis with other cancers as competing events. We tested if there was difference in familial risks associated with LCa-1 and LCa-2 diagnoses in FDRs in two ways: 1) the full model (the Poisson regression model above), 2) full model with additional adjustment for age at diagnosis of LCa in FDRs (5-year group) and family relationship. The results could indicate whether the different age at diagnosis of LCa in FDRs and family relationship contributed the potential different familial risk. To reveal the familial aggregation mainly caused by genetic component for lung cancer, we analyzed the familial risk by excluding individuals with diagnosis of COPD, who were assumed as heavy smokers. A two-sided *p* value less than 0.05 was considered to be statistically significant. All the statistical analyses were performed with the use of SAS statistical software, version 9.4 (SAS Institute) and R (version 3.6.2).

## Results

**Fig. 2:**
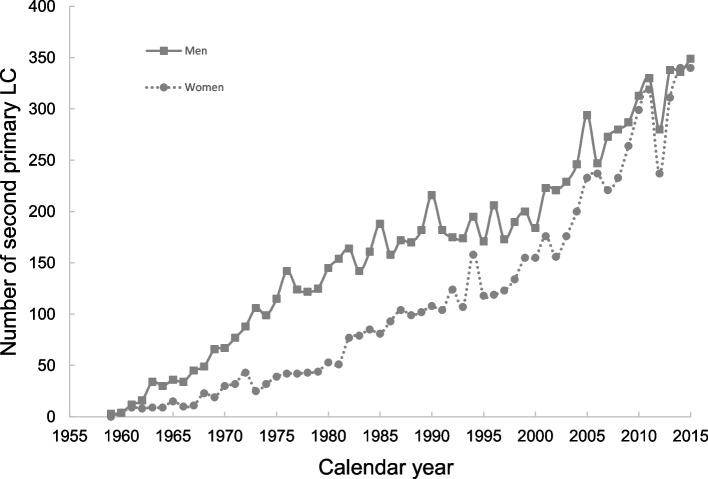
Temporal trend of LCa-2 stratified by gender in Swedish Cancer Register among 246,021 male and 254,307 female cancer patients. LCa-2, lung cancer as a second primary malignancy

The median (IQR) diagnostic age for LCa-1 and LCa-2 in the cancer registry was 69 (62–76) and 73 (67–79), respectively. The annual number of LCa-2 has been increasing in both men and women (Fig. 2). Over the years more men were diagnosed with LCa-2, although in the last decade the number for both sexes were similar.

**Fig. 3:**
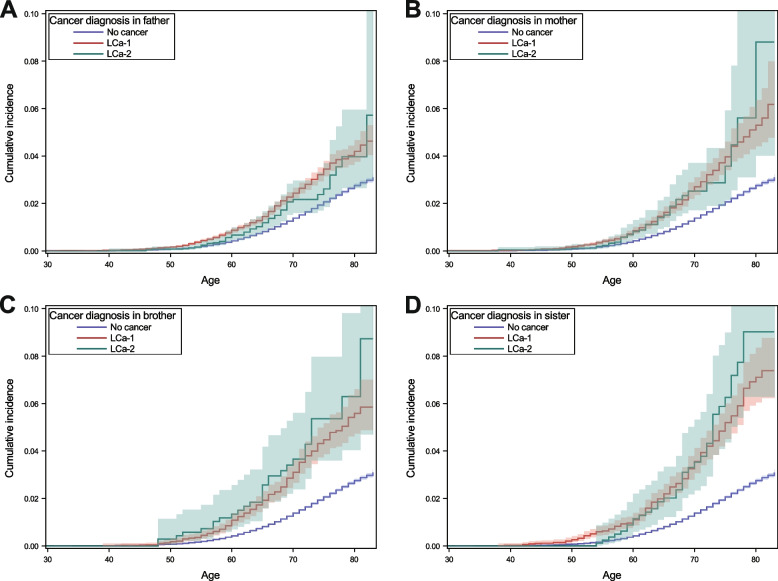
Cumulative incidence of lung cancer (LCa) in the offspring generation (born from 1932) of Swedish Cancer Register with a family history of LCa-1 or LCa-2 in father (**A**), mother (**B**), brother (**C**) and sister (**D**). A total of 71,873 individuals had father, 41,915 had mother, 7,032 had one brother and 7,045 had one sister diagnosed with LCa-1. For LCa-2, the corresponding numbers were 8,309 (father), 5,609 (mother), 711 (brother) and 861 (sister). The shading band is the 95% confidence interval of the cumulative incidence. LCa-1, lung cancer as a first primary malignancy, LCa-2, lung cancer as a second primary malignancy

A total of 8,171,958 individuals in the offspring generation were identified at risk of first LCa. The maximum age for the offspring in 2015 was 84 years, whereas the age for the parents was not limited. After removing 2,439,821 individuals who did not reach the inclusion criteria (see study population selection in Supplementary Fig. 1), 5,588,782 individuals remained whose FDRs were not diagnosed with cancer (reference population), 127,865 whose FDRs were diagnosed with LCa-1 and 15,490 whose FDRs were diagnosed with LCa-2. LCa risk among FDRs of patients diagnosed with LCa-1 (RR, 1.96, 95%, 1.85–2.07) and LCa-2 (1.89, 1.62–2.21) was similar (Table 1). A higher risk was observed for individuals whose FDRs were diagnosed with LCa at a younger age. For both LCa-1 and LCa-2, familial risk was strongly associated with sibling family history compared to parental family history. For each family relationship, the cumulative incidence across age overlapped between family history of LCa-1 and LCa-2 (Fig. 3). The median time between the first primary cancer to LCa-2 in FDRs was 74 (26–152) months. In the period- stratified analysis (Table 1), the LCa risk was 1.53 (1.10–2.12) when LCa-2 was diagnosed within 26 months after first primary cancer. It increased to 2.16 (1.62–2.90) when the time was between 74 to 154 months. We did not observe any significant difference in familial risks associated with LCa-1 and LCa-2 diagnoses in FDRs (Table 1, last two columns). Among individuals without diagnosis of COPD (Supplementary Table 1), the association between LCa risk and family history of LCa-1 or LCa-2 were similar to the results in the main analysis.

**Table 1:** Lung cancer risk among individuals who had one first-degree relative affected by LCa-1 or LCa-2

Category	Cancer diagnosis in FDR								Comparison^c^ (LCa-1 vs. LCa-2)	
	LCa-1				LCa-2					
	Age at diagnosis of LCa in FDR	N^a^	RR^b^	95%CI	Age at diagnosis of LCa in FDR	N^a^	RR^b^	95%CI	P1	P2
Overall	66 (59–73)	1474	1.96	1.85–2.07	71 (65–77)	161	1.89	1.62–2.21	0.56	0.93
Age in relative with LCa										
≤ 60 years old	55 (49.5–58)	321	2.34	2.09–2.62	56 (51–58)	15	2.55	1.54–4.23	0.70	0.93
> 60 years old	70 (65–76)	1153	1.87	1.76–1.99	72 (68–78)	146	1.84	1.56–2.17	0.69	0.90
Type of family history										
Only father	67 (60–74)	707	1.74	1.61–1.88	73 (66–78)	57	1.40	1.08–1.81	0.07	0.30
Only mother	66 (58–73)	321	1.95	1.74–2.18	70 (63–77)	39	1.81	1.33–2.49	0.51	0.71
Only brother	63 (56–69)	215	2.21	1.93–2.53	68 (61–72)	28	2.84	1.96–4.11	0.35	0.34
Only sister	62 (55–68)	236	2.64	2.33–3.01	67 (61–77)	37	2.90	2.10–4.00	0.60	0.53
Time between first primary cancer and LCa-2 in FDR										
< 26 months					70 (63–75)	36	1.53	1.10–2.12		
26–73 months					71 (65–77)	36	1.85	1.34–2.57		
74–152 months					71 (65–77)	46	2.16	1.62–2.90		
> 152 months					72 (66–78)	43	2.07	1.54–2.78		

*LCa* lung cancer, *LCa-1* Lung cancer as a first primary malignancy, *LCa-2 *Lung cancer as a second primary malignancy, *FDR* First-degree relative, *RR* Relative risk, *95%CI* 95% confidence interval

^a^N, number of LCa cases diagnosed during the follow-up

^b^RR was estimated from Poisson regression using individuals without cancer family history as the reference. The covariates adjusted in the model included age groups (5 years), periods (5 years), hospitalization due to COPD, alcoholism and obesity, socioeconomic status (blue-collar worker, white-collar worker, farmer, private business, professional, or other/unspecified) and place of residence (big cities, northern Sweden, southern Sweden and unspecific)

^c^Comparison of familial risks associated with family history of LCa-1 and LCa-2 with Poisson regression. P1 is the *p* value for the comparison when the adjusted covariates were same as main analysis (above). P2 is the *p* value for the comparison when age at diagnosis of LCa in FDR (5-year group) and family relationship were additionally adjusted

When stratifying the first primary cancer site before LCa-2 in FDRs (Table 2, sites are ordered by number of LCa cases), we observed high familial risks for first primary cancer in the ovary (4.45, 1.85–10.7), nervous system (3.49, 1.45–8.38), non-Hodgkin lymphoma (3.13, 1.57–6.27), UAT (2.83, 1.78–4.49) and cervix (2.55, 1.41–4.61).

**Table 2:** Lung cancer risk among individuals with a family history of LCa-2 stratified by different first primary cancer diagnosed in their relatives

Site of first primary cancer before LCa-2 in relatives	N^a^	RR^b^	95%CI	
Prostate	22	**1.55**	1.02	2.35
Breast	21	**1.94**	1.27	2.98
Upper aerodigestive tract	18	**2.83**	1.78	4.49
Colorectum	14	**1.77**	1.05	3.00
Bladder	13	1.22	0.71	2.11
Cervix	11	**2.55**	1.41	4.61
Skin	9	1.79	0.93	3.45
Non-Hodgkin lymphoma	8	**3.13**	1.57	6.27
Kidney	6	2.06	0.92	4.58
Ovary	5	**4.45**	1.85	10.7
Nervous system	5	**3.49**	1.45	8.38
Leukemia	5	1.79	0.74	4.30
Stomach	< 5	1.03	0.15	7.33
Small intestine	< 5	3.81	0.95	15.2
Liver	< 5	3.93	0.98	15.7
Nose	< 5	3.43	0.48	24.4
Endometrium	< 5	2.22	0.83	5.92
Female genital	< 5	2.44	0.34	17.3
Male genital	< 5	2.15	0.30	15.3
Melanoma	< 5	0.95	0.31	2.95
Thyroid	< 5	2.38	0.59	9.51
Endocrine gland	< 5	1.70	0.55	5.26
Connective tissue	< 5	3.54	0.50	25.1
Hodgkin lymphoma	< 5	1.44	0.20	10.2
Cancer of unknown primary	< 5	0.83	0.12	5.86

*LCa* Lung cancer, *LCa-1* Lung cancer as a first primary malignancy, *LCa-2* Lung cancer as a second primary malignancy, *RR* Relative risk, *95%CI* 95% confidence interval

^a^N, number of LCa cases diagnosed during the follow-up

^b^RR was estimated from Poisson regression using individuals without cancer family history as the reference. The covariates adjusted in the model included age groups (5 years), periods (5 years), hospitalization due to COPD, alcoholism and obesity, socioeconomic status (blue-collar worker, white-collar worker, farmer, private business, professional, or other/unspecified) and place of residence (big cities, northern Sweden, southern Sweden and unspecific). Significant RRs are in bold

## Discussion

The principal finding of this nationwide cohort study is that familial RR of LCa was associated with the diagnosis of LCa-2 in FDR similarly to that with LCa1- in FDR. The risk of LCa was high when early-onset LCa-2 was diagnosed in FDRs and when siblings were affected by LCa-2. High risks were observed for first primary cancer in the ovary, nervous system, upper aerodigestive tract and cervix, and in non-Hodgkin lymphoma.

We observed that the number of LCa-2 in the last decade has become comparable between men and women, which is consistent with the increasing trend of total LCa incidence among women in Sweden [16]. This is probably due to the relatively higher prevalence of smoking among women today in Sweden where the proportion of female smokers is larger among people aged 16–84 as calculated between 2006 to 2015 [17]. In a Chinese study LCa patients with prior cancer were observed to harbor pathogenic or likely-pathogenic mutations for LCa with higher frequency [18]. The rising number of LCa-2 justified the necessity of our study on familial risk of LCa in relatives.

The overall risk of LCa is similar regardless of the order of LCa in FDRs i.e., first or second primary. A family history of LCa-2 shared similar features with LCa-1; risk was high when early-onset LCa was diagnosed in FDRs and when siblings were affected by LCa. It is well-accepted that an earlier age at diagnosis of cancer is indicative of a higher likelihood of inherited risk [4]. The lower risk with a parental family history of LCa-2 compared to that with LCa-1 is consistent with the late onset of LCa-2 in parents (median age at diagnosis in father and mother: 73 and 70) compared to LCa-1 (67 and 66). Even though the age at diagnosis of LCa-2 in brother was older than LCa-1, the LCa risk for having a sibling history of LCa-2 was still higher than having that of LCa-1. This could be related to some unknown genetic or environmental factors shared by the brothers, which needs further investigation. When LCa-2 was diagnosed within 26 months after the first primary cancer, the observed lower familial risk may be attributed to the intensive medical surveillance after the first cancer diagnosis. This is supported by the large proportion of early stage-LCa (stage I/ II) among patients with LCa-2 diagnosed within 26 months (35.8%, data not shown in the results) compared to the relatively small proportion in the period 26–73 months (24.2%), 74–152 months (28.1%) and > 152 months (20.6%).

In the site-stratified analysis, we identified increased risk in individuals whose FDR was diagnosed with LCa-2 after some specific types of first primary cancer. Many of these are smoking-related cancers [19], such as cancers of upper aerodigestive tract, cervix, kidney and bladder. The varied LCa risk for different first primary cancer in family member suggests that first primary cancer site before LCa-2 in FDR is an important predictor for LCa risk. However, the case number for many cancers is small, thus larger multi-nation studies are needed to provide accurate risk estimates.

When dealing with multiple primary cancers, it is difficult to differentiate between the new malignancy and metastases from existing cancer. In Sweden, the diagnosis of multiple primary cancers follows the IARC/IACR multiple cancer coding rules [20]. Previously an ad hoc study showed 98% diagnostic accuracy of second neoplasms in the cancer registry; no recorded second primary cancer was found to be a metastasis [21]. Some specific strengths of this studies are as follows: The registry-based data on family history of LCa can avoid information bias. In addition, the nationwide scale provided our study with large sample size, enabling a reliable risk estimation. We used several stratification analyses to describe the feature of the family history of LCa-2, generating evidence for a better precise LCa prevention. We identified some smoking-related cancers with high LCa risk if these cancers are diagnosed as first primary in FDR before a LCa-2. We are also aware that for some cancers with poor survival such as pancreatic cancer, there might be under reporting on LCa-2. We did not observe more smoking-related cancers among the first primaries, possibly due to the strict inclusion criteria that individuals could only have one family member diagnosed with LCa-2. Thus, many families where multiple family members were diagnosed with smoking-related cancers, have been filtered out. Information on smoking, physical activity and alcohol consumption are not available in this nationwide register studies. However, we adjusted the hospitalization due to COPD, obesity and alcoholism, which may help reduce the possible confounding. Similarly, adjustment for socioeconomic status and place of residence was also to control some occupational or environmental exposure that can increase LCa risk, such as asbestosis and radon. We acknowledge that these variables cannot replace the effect of the original variables. We also emphasize that study with larger sample size is needed for better assessment of site-specific risk of LCa based on first primary cancer in FDR as well as risk from other type of family relationship like half sibling.

## Conclusion

To the best of our knowledge, this is the first to study the familial risk of LCa among individuals whose FDRs were diagnosed with LCa-2. This similar risk among individuals with family history of LCa-1 or LCa-2 suggests that they can be managed similarly regarding LCa prevention. Compared to family history of LCa-1, LCa risk associated with family history of LCa-2 can be better predicted based on the cancer diagnosed before LCa-2 in FDRs.

## Supplementary Information

**Additional file 1: Supplementary Figure 1**. Selection of study population. **Supplementary Table 1.** Association between lung cancer risk and family history of LCa-1 or LCa-2 among individuals without diagnosis of chronic obstructive pulmonary disease.

## Data Availability

The data that support the findings of this study are available from Lund University but restrictions apply to the availability of these data, which were used under license for the current study and so are not publicly available. Any request regarding the data from this study should go to the last author (J.J.).
