# Supplementary material for: Familial risk associated with lung cancer as a second primary malignancy in first-degree relatives
Source: BMC Cancer. 2022 Oct 12;22:1057. doi: 10.1186/s12885-022-10149-7 (PMC9555112; doi:10.1186/s12885-022-10149-7)
Supplement: Supplementary file 1 — Additional file 1: Supplementary Figure 1. Selection of study population. Supplementary Table 1. Association between lung cancer risk and family history of LCa-1 or LCa-2 among individuals without diagnosis of chronic obstructive pulmonary disease. [file 12885_2022_10149_MOESM1_ESM.pdf]

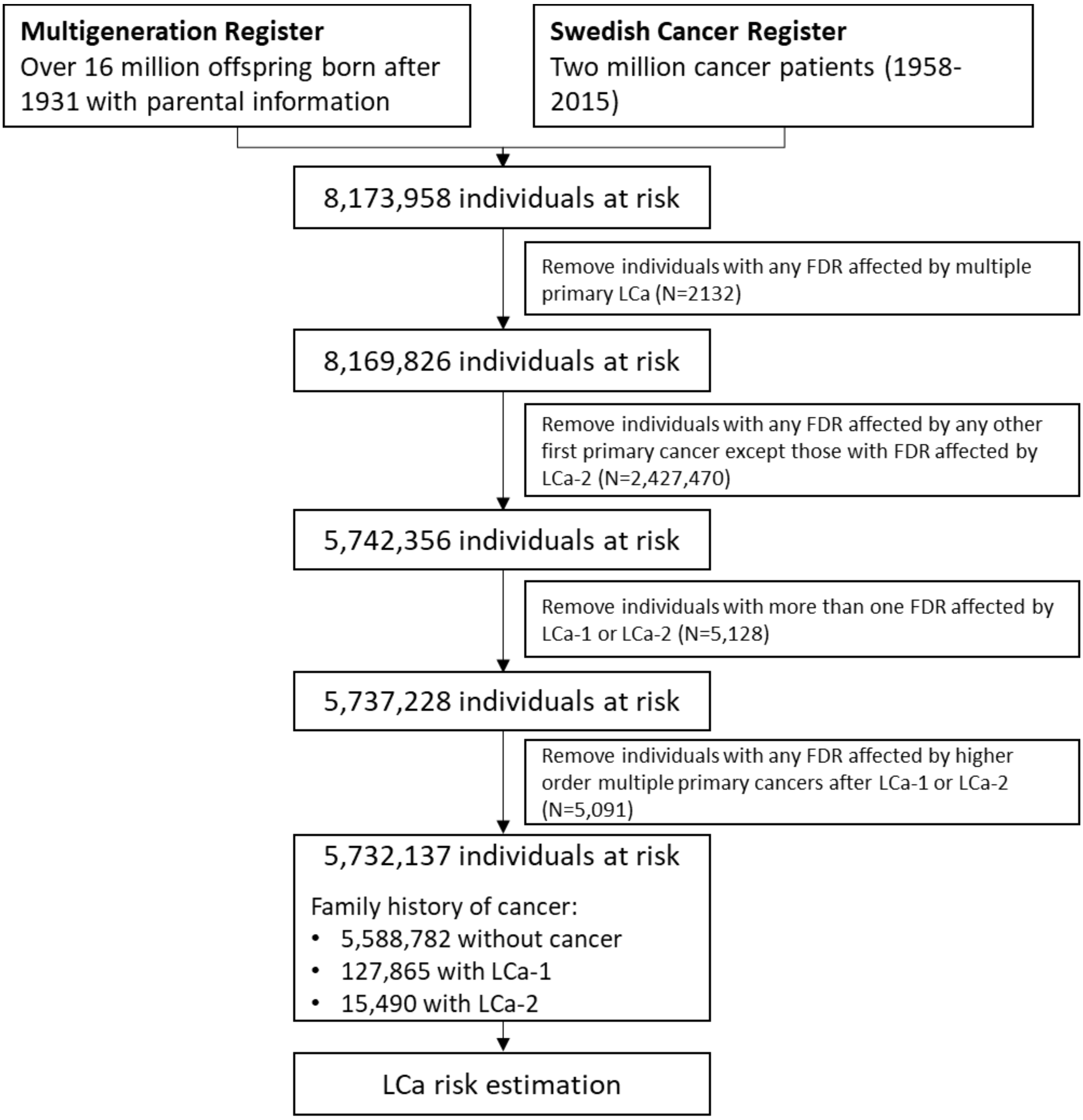

Supplementary Figure 1 Selection of study population

Supplementary Table 1. Association between lung cancer risk and family history of LCa-1 or LCa-2 among individuals without diagnosis of chronic obstructive pulmonary disease

	Cancer diagnosis in FDR							
Category	LCa-1				LCa-2			
	Age at diagnosis of LCa in FDR	N [a]	RR [b]	95%CI	Age at diagnosis of LCa in FDR	N [a]	RR [b]	95%CI
Overall	66 (59-73)	1255	1.98	1.87-2.10	71 (65-77)	131	1.84	1.55-2.19
Age in relative with LCa								
≤60 years old	55 (50-58)	274	2.37	2.10-2.68	56 (51-58)	13	2.57	1.49-4.43
>60 years old	70 (65-76)	981	1.90	1.78-2.03	72 (68-78)	118	1.79	1.49-2.14
Type of family history								
Only father	67 (60-74)	603	1.77	1.63-1.92	73 (67-78)	50	1.44	1.09-1.91
Only mother	66 (58-73)	274	1.97	1.74-2.22	69 (63-76)	31	1.71	1.20-2.43
Only brother	63 (56-69)	178	2.22	1.92-2.58	68 (61-72)	24	2.97	1.99-4.43
Only sister	62 (55-68)	200	2.76	2.40-3.18	66 (61-71)	26	2.53	1.72-3.72
Time between first primary cancer and LCa-2 in FDR								
<26 months					70 (63-75)	32	1.54	1.09-2.18
26-73 months					71 (65-77)	27	1.70	1.17-2.49
74-152 months					71 (65-77)	38	2.27	1.65-3.12
>152 months					72 (65-78)	34	1.91	1.36-2.67

[a], N, number of LCa cases diagnosed during the follow-up.

[b], RR was estimated from Poisson regression using individuals without cancer family history as the reference. The covariates adjusted in the model included age groups (5 years), periods (5 years), hospitalization due to alcoholism and obesity, socioeconomic status (blue-collar worker, white-collar worker, farmer, private business, professional, or other/unspecified) and place of residence (big cities, northern Sweden, southern Sweden and unspecific).

LCa, lung cancer, LCa-1, lung cancer as a first primary malignancy, LCa-2, lung cancer as a second primary malignancy, FDR, first-degree relative, RR, relative risk, 95%CI, 95% confidence interval.
